# GRACE scores or high-sensitivity troponin for timing of coronary angiography in non-ST-elevation acute coronary syndromes

**DOI:** 10.1007/s00392-023-02258-5

**Published:** 2023-07-08

**Authors:** Alexander Jobs, Jasper Boeddinghaus, Johannes Tobias Neumann, Alina Goßling, Nils A. Sörensen, Raphael Twerenbold, Thomas Nestelberger, Pedro Lopez-Ayala, Maria Rubini Gimenez, Oscar Miro, Luca Koechlin, Natacha Buergin, Hans-Josef Feistritzer, Jean-Philippe Collet, Deepak L. Bhatt, Christopher B. Granger, Stefan Blankenberg, Steffen Desch, Christian Mueller, Dirk Westermann, Holger Thiele

**Affiliations:** 1 Department of Internal Medicine/Cardiology and Leipzig Heart Institute, Heart Center Leipzig at University of Leipzig, Strümpellstr. 39, 04289 Leipzig, Germany; 2 German Centre for Cardiovascular Research (DZHK), Partner Site Hamburg/Luebeck/Kiel, Hamburg, Germany; 3 Cardiovascular Research Institute Basel (CRIB) and Department of Cardiology, University Hospital Basel, University of Basel, Basel, Switzerland; 4 BHF Centre for Cardiovascular Science, University of Edinburgh, Edinburgh, Scotland, UK; 5 Department of Cardiology, University Heart & Vascular Center Hamburg, University Medical Center Hamburg-Eppendorf, Hamburg, Germany; 6 Department of Epidemiology and Preventive Medicine, School of Public Health and Preventive Medicine, Monash University, Melbourne, Australia; 7 Emergency Department, Hospital Clinic, Barcelona, Catalonia Spain; 8 Sorbonne Université, ACTION Group, INSERM UMRS 1166, Hôpital Pitié-Salpêtrière (AP-HP), Institut de Cardiologie, Paris, France; 9 Brigham and Woman’s Hospital, Harvard Medical School, Boston, USA; 10 Duke University Medical Center, Durham, NC USA; 11 Clinic for Cardiology and Angiology, University Heart Center Freiburg - Bad Krozingen, Freiburg and Bad Krozingen, Germany

**Keywords:** Acute coronary syndrome without persistent ST-segment elevation, Non-ST-segment myocardial infarction, GRACE risk score, Risk stratification, High-sensitivity cardiac troponin

## Abstract

**Background:**

The GRACE risk score is generically recommended by guidelines for timing of invasive coronary angiography without stating which score should be used. The aim was to determine the diagnostic performance of different GRACE risk scores in comparison to the ESC 0/1 h-algorithm using high-sensitivity cardiac troponin (hs-cTn).

**Methods:**

Prospectively enrolled patients presenting with symptoms suggestive of myocardial infarction (MI) in two large studies testing biomarker diagnostic strategies were included. Five GRACE risk scores were calculated. The amount of risk reclassification and the theoretical impact on guideline-recommended timing of invasive coronary angiography was studied.

**Results:**

Overall, 8,618 patients were eligible for analyses. Comparing different GRACE risk scores, up to 63.8% of participants were reclassified into a different risk category. The proportion of MIs identified (i.e., sensitivity) dramatically differed between GRACE risk scores (range 23.8–66.5%) and was lower for any score than for the ESC 0/1 h-algorithm (78.1%). Supplementing the ESC 0/1 h-algorithm with a GRACE risk score slightly increased sensitivity (*P* < 0.001 for all scores). However, this increased the number of false positive results.

**Conclusion:**

The substantial amount of risk reclassification causes clinically meaningful differences in the proportion of patients meeting the recommended threshold for pursuing early invasive strategy according to the different GRACE scores. The single best test to detect MIs is the ESC 0/1 h-algorithm. Combining GRACE risk scoring with hs-cTn testing slightly increases the detection of MIs but also increases the number of patients with false positive results who would undergo potential unnecessarily early invasive coronary angiography.

**Graphical abstract:**

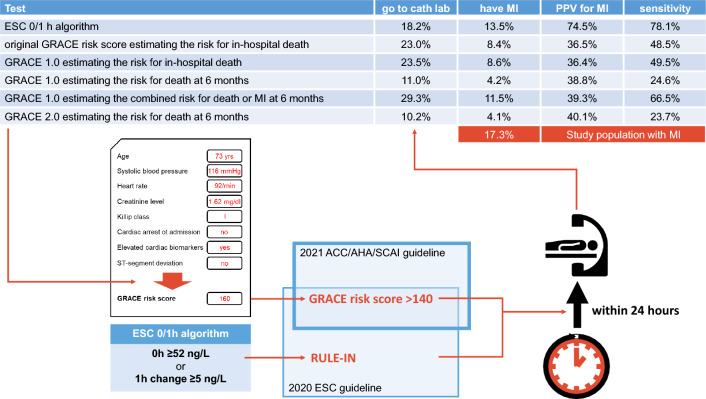

## Introduction

Based on the Global Registry of Acute Coronary Events (GRACE), several risk scores have been developed to predict ischemic risk in patients presenting with an acute coronary syndrome (ACS). The first risk score was published in 2003 and estimated the risk for in-hospital death [1]. It was developed using 11,389 patients enrolled from 1999 to 2001 in the GRACE registry. Subsequently, several other scores have been developed based on the till 2009 enrolling GRACE registry (i.e., GRACE 1.0 and 2.0) [2, 3]. These scores were derived from different subsets of the GRACE population and estimate the risk for death after a longer follow-up or the combined risk for death or myocardial infarction (MI). All GRACE risk scores calculated at presentation use the same eight variables. However, weighting of these variables differs between the scores. While the original GRACE score reported a single risk score calculated by means of a nomogram, GRACE 1.0 and GRACE 2.0 are web calculators estimating the risk for different outcomes and display the result of multiple scores [1–3].

American and European guidelines on the management of non-ST-segment elevation acute coronary syndrome (NSTE-ACS) as well as myocardial revascularization recommend the GRACE risk score not only for risk prediction but also for specific treatment decisions. It is one of the criteria indicating high ischemic risk (cutoff > 140) for which an early invasive strategy within 24 h is recommended across all major guidelines [4–7]. While the American 2021 guidelines for coronary artery revascularization specifically recommend the *GRACE 2.0 estimating the risk for death at 6 months* for timing of invasive coronary angiography [7], other guidelines do not specifically indicate which specific GRACE risk score should be used [4–6]. In fact, the evidence from the TIMACS trial was based on the original GRACE risk score [8]. More importantly, American and European guidelines relevantly differ with respect to criteria for an early invasive strategy. Whereas European Society of Cardiology (ESC) guidelines include the ESC 0/1 h- or 0/2 h- algorithm using high-sensitivity cardiac troponin (hs-cTn) and the GRACE risk score > 140 criteria, American guidelines do not apply this hs-cTn criterion for an early invasive strategy within 24 h. Independently from the originally intended purpose, both tools are applied as risk stratification tools to identify high-risk patients most likely benefiting from an early invasive strategy.

Applying different GRACE risk scores to the same patient will lead to different point scores (see case-vignette in the supplement). This might influence treatment decisions in specific cases. The aim of this analysis was, therefore, (1) to study the proportion of risk reclassification with other GRACE risk scores than the *original GRACE risk score* (applying recommended cutoffs), (2) to determine the impact of the GRACE risk score choice on guideline-recommended early invasive strategy (i.e., invasive coronary angiography within 24 h), and (3) to determine if the ESC 0/1 h-algorithm using hs-cTn or GRACE risk scores perform better for early invasive strategy selection in a large population of patients presenting to the emergency department (ED) with symptoms suggestive of an NSTE-ACS.

## Methods

### Study population

This analysis is based on pooled patient-level data of BACC (Biomarkers in Acute Cardiac Care; NCT02355457) [9] and APACE (Advantageous Predictors of Acute Coronary Syndromes Evaluation; NCT00470587) observational studies [10, 11]. In both studies, patients presenting to the ED with symptoms suggestive of MI (such as acute chest discomfort) with an onset or peak within the last 12 h and an age ≥ 18 were recruited. For this analysis, patients with STEMI or Type 4 MI as well as patients with an unknown diagnosis after final adjudication were excluded. For the calculation of the ESC 0/1 h-algorithm, patients with missing 1 h hs-cTnT concentrations were further excluded (Online Fig. 1).

All patients underwent routine clinical assessment including medical history, physical examination, standard blood tests including serial measurements of local hs-cTnT, 12-lead electrocardiogram (ECG), chest radiography (if requested), continuous ECG rhythm monitoring, and pulse oximetry. Management of patients was left to the discretion of the attending physician.

Two independent cardiologists in a dedicated core laboratory performed central adjudication of the final diagnosis using all available medical records obtained during clinical care as well as study-specific assessments. A third cardiologist was consulted in cases of disagreement on the final diagnosis. Further information is provided in the Online Supplement.

### GRACE risk scores

GRACE risk score models were calculated by adding the points assigned to the following eight variables recorded at hospital admission: age, heart rate, systolic blood pressure, serum creatinine, Killip class, cardiac arrest, ST-segment deviation, and elevated cardiac enzymes/markers. Except for the Killip class, all variables were prospectively recorded in APACE and BACC. We approximated the Killip class for this analysis using the New York Heart Association (NYHA) functional class and the systolic blood pressure as outlined in Online Table 2. The binary variable elevated cardiac enzymes/markers was set to yes if the first determined hs-cTnT was > 14 ng/l. We calculated the following GRACE risk scores by means of R scripts (Online Appendix):

- Original GRACE risk score estimating the risk for in-hospital death,
- GRACE 1.0 estimating the risk for in-hospital death,
- GRACE 1.0 estimating the risk for death at 6 months,
- GRACE 1.0 estimating the combined risk for death or myocardial infarction at 6 months, and
- GRACE 2.0 estimating the risk for death at 6 months.

In clinical routine, the original GRACE risk score can be calculated using a nomogram depicted in the original publication, whereas the GRACE 1.0 and GRACE 2.0 scores can be calculated using web calculators or alternatively applications for mobile devices.

### Measurements of hs-cTnT

Blood samples (plasma and serum) were collected at the time of the patients’ presentation to the ED and after 1 h. Levels of hs-cTnT were determined on the Elecsys (Roche Diagnostics, Rotkreuz, Switzerland). Further details are provided in the Online Supplement.

### ESC 0/1 h-algorithm

The purpose of the 0/1 h-algorithm is to triage patients presenting with symptoms suggesting NSTEMI toward rule-out, observe, and rule-in. To do so, the algorithm processes the hs-cTn levels determined from two subsequent blood withdrawals. The ESC 0/1 h-algorithm compares the absolute change from presentation to 1 h thereafter. The cutoff values for the absolute change used by the ESC 0/1 h-algorithms are assay specific and were derived in previous diagnostic studies [5, 6]. For this analysis, hs-cTnT was used.

### Outcome measures and follow-up

The diagnostic endpoint was NSTEMI (type 1 and 2 MI) during index hospitalization. The prognostic endpoints were in-hospital death, death at 6 months, and death during the entire follow-up. Patients were regularly contacted up to 12 months after discharge by telephone calls or in written form. Additionally, information regarding death during follow-up was obtained from the patient’s hospital notes, the family physician’s records, and the national registries on mortality.

### Statistical analyses

Missing data were handled by multiple imputation by chained equations (ten imputed data sets; R package *mice*). Multiple imputation was performed separately for the patients from APACE and BACC. All variables used for the multiple imputation are shown in Online Table 3. Patients were grouped according to the GRACE risk score group (i.e., low, intermediate, or high risk) to show patient characteristics. Continuous variables are shown as median (25th and 75th percentile) and were compared by the Kruskal–Wallis test. Binary variables are shown as absolute numbers (percentages) and the $\chi^{2}$ test was used for comparison. To answer aim (1), the distributions of patients reclassified by GRACE risk scores other than the *original GRACE risk score* were determined. The risk categories (low, intermediate, or high risk) with cutoffs drawn from the *original GRACE risk score* (< 108, 108–140, or > 140, respectively) were used for this.

Sensitivity, specificity, and positive predictive value (PPV) together with their 95% confidence intervals (CI) were calculated to determine the diagnostic performances of the ESC 0/1 h-algorithm and GRACE risk scores (aim 2 and 3). The McNemar test was used to compare sensitivity and specificity and the test of Kosinski to compare the PPV between diagnostic tests [12]. The impact on the diagnostic performance by combining the ESC 0/1 h-algorithm with a GRACE risk score to one test was also determined. Further analyses are described in the Online Supplement.

All statistical analyses were performed using R version 4.0.3 (R Foundation for Statistical Computing).

## Results

From April 2006 to April 2018, 8,766 patients were enrolled either in the APACE or the BACC studies. In total, 148 patients with the final diagnosis of STEMI or type 4 MI as well as missing information about the final diagnosis were excluded. This led to a final analysis population of 8,618 patients (Online Fig. 2).

The proportion of missing data in variables necessary for the calculation of the GRACE risk score was low (Online Table 4). The variables with the most missing observations were the Killip class and ST-segment change (0.8% each). Overall, 187 of 8,618 (2.2%) patients had missing data on at least one variable. Only minor differences in baseline characteristics exist between patients without and with missing data (Online Table 5). Patients with missing data did not have a higher probability for the primary diagnostic endpoint during index hospitalization (15.0 versus 14.1%; *p* = 0.82) or a higher risk to die during follow-up (9.3 versus 6.8%; *p* = 0.29).

Baseline characteristics in the overall population and in subgroups according to risk categories based on the *original GRACE risk score* are shown in Table 1. As expected, patients in higher risk categories were older, had a higher heart rate, lower systolic blood pressure, higher Killip class, higher serum creatinine, and more often cardiac arrest, ST-segment deviation as well as elevated cardiac enzymes/markers.

**Table 1:** Patient characteristics

	All (*n* = 8618)	Low (*n* = 4516)	Intermediate (*n* = 2120)	High (*n* = 1982)	*p*
Male, %	5658 (65.7)	3078 (68.2)	1352 (63.8)	1228 (62.0)	< 0.001
BMI, kg/m^2^	26.4 (23.8, 29.6)	26.3 (23.6, 29.6)	26.8 (24.2, 29.9)	26.2 (23.7, 29.4)	< 0.001
GRACE variables					
Age, years	62.0 (49.0, 74.0)	51.0 (42.0, 59.0)	70.0 (61.4, 76.0)	78.0 (72.0, 83.0)	< 0.001
Heart rate, min^−1^	76.0 (66.0, 89.0)	76.0 (66.0, 87.0)	75.0 (65.0, 88.0)	80.0 (69.0, 97.0)	< 0.001
Systolic blood pressure, mmHg	142.0 (127.0, 158.0)	143.0 (129.0, 160.0)	145.0 (130.0, 159.0)	134.0 (118.0, 151.0)	< 0.001
Killip class					< 0.001
I	6875 (79.8)	4268 (94.5)	1660 (78.3)	947 (47.8)	
II	1463 (17.0)	235 (5.2)	406 (19.2)	822 (41.5)	
III	263 (3.1)	13 (0.3)	54 (2.5)	196 (9.9)	
IV	17 (0.2)	0 (0)	0 (0)	17 (0.9)	
Creatinine, µmol/l	79.0 (67.0, 94.6)	74.0 (64.0, 85.7)	80.4 (69.0, 97.0)	96.0 (78.0, 121.0)	< 0.001
ST-segment deviation, %	1756 (20.4)	292 (6.5)	514 (24.2)	950 (47.9)	< 0.001
Hs-cTnT (Roche) > 14 ng/L, %	2782 (32.3)	381 (8.4)	840 (39.6)	1561 (78.8)	< 0.001
Cardiac arrest, %	36 (0.4)	0 (0)	4 (0.2)	32 (1.6)	< 0.001
Medical history					
History of myocardial infarction, %	1807 (21.0)	602 (13.3)	515 (24.3)	690 (34.8)	< 0.001
History of coronary artery disease, %	2809 (32.6)	890 (19.7)	866 (40.8)	1,053 (53.1)	< 0.001
Hypertension, %	5272 (61.2)	2045 (45.3)	1565 (73.9)	1662 (83.9)	< 0.001
Hyperlipoproteinemia, %	3742 (43.4)	1469 (32.5)	1113 (52.5)	1160 (58.5)	< 0.001
Current smoker, %	2078 (24.2)	1448 (32.2)	386 (18.3)	244 (12.4)	< 0.001
Peripheral artery disease, %	470 (5.5)	87 (1.9)	140 (6.6)	243 (12.3)	< 0.001
Stroke, %	478 (5.5)	112 (2.5)	155 (7.3)	211 (10.7)	< 0.001
Atrial fibrillation, %	760 (8.8)	96 (2.1)	191 (9.0)	473 (23.9)	< 0.001
Laboratory findings					
hs-TnT 0 h, ng/l	8.0 (4.0, 20.0)	5.0 (3.0, 8.0)	12.0 (7.0, 22.2)	26.0 (16.0, 59.0)	< 0.001
eGFR, ml/min/1.73 m^2^	85.1 (66.4, 99.2)	96.7 (84.4, 107.2)	78.0 (63.5, 89.7)	59.3 (44.2, 77.0)	< 0.001
ECG findings					
ST-segment depression, %	823 (9.6)	95 (2.1)	237 (11.2)	491 (25.1)	< 0.001
T-wave inversion, %	987 (11.5)	150 (3.3)	299 (14.2)	538 (27.5)	< 0.001
No significant ECG changes, %	6,812 (79.7)	4,199 (93.6)	1597 (75.7)	1016 (52.0)	< 0.001

Descriptive patient characteristics are based on the first imputed data set, while statistical testing for group difference is based on the multiple imputed dataset. Values are median (interquartile range) or n (%). Estimated glomerular filtration rate was calculated using CKD-EPI (chronic kidney disease epidemiology collaboration) formula

*BMI* body mass index, *ECG* electrocardiogram, *eGFR* estimated glomerular filtration rate, *hs-cTnI* high-sensitivity cardiac troponin I, *hs-cTnT* high-sensitivity cardiac troponin T

### GRACE risk scores

Weighting of variables differed between scores (Online Table 1). The most noticeable differences in the GRACE risk scores are that the heart rate does not contribute to the score in the *GRACE 1.0 estimating the combined risk for death or myocardial infarction at 6 months* and that input ranges of the GRACE 2.0 differ considerably to that of the *original GRACE risk score* and the GRACE 1.0 scores. The range of the final scores differ considerably; while it is in the range of 1–306 for the *GRACE 1.0 estimating the risk for death at 6 months*, it is 2–423 for the *GRACE 1.0 estimating the combined risk for death or myocardial infarction at 6 months*.

### Risk reclassification

As compared to the *original GRACE risk score*, other GRACE risk scores reclassified a substantial number of patients. The highest reclassification rate was observed for the *GRACE 1.0 and 2.0 score estimating the risk for death within 6 months*. These two scores downgraded > 60% of patients in the intermediate-risk group to the low-risk group as well as > 50% of patients in the high-risk group to the intermediate-risk group (Table 2).

**Table 2:** Reclassification of risk by GRACE risk scores other than the original Granger score

	Risk category	Original GRACE risk score estimating the risk for in-hospital death		
		Low	Intermediate	High
Score, outcome	Percent of patients	52.4	24.6	23.0
GRACE 1.0, in-hospital death	Low		6.5	0
	Intermediate	0.1		0
	High	0	2.2	
GRACE 1.0, death within 6 months	Low		63.8	0.5
	Intermediate	0		51.8
	High	0	0	
GRACE 1.0, death or MI within 6 months	Low		18.7	0.2
	intermediate	5.7		4.1
	High	0.002	29.6	
GRACE 2.0, death within 6 months	Low		67.9	1.8
	Intermediate	0.02		53.7
	High	0	0	

Percent of patients reclassified by GRACE risk scores other than the original GRACE risk score estimating the risk for in-hospital death, which is used as reference score

The upper part of the table shows the percent of patients categorized as low, intermediate, or high risk by the reference score. The percentage of patients reclassified by others GRACE risk scores is shown beneath. The presented data are based on the imputed data. For example, the reference score categorized 24.6% of patients to be at intermediate risk. From these 24.6% of patients in the intermediate-risk group, 63.8% are downclassified to the low-risk group by GRACE 1.0 estimating the risk for death at 6 months

### Final diagnosis and clinical outcome

The final diagnosis at the end of the index hospitalization was type 1 MI in 1,109 of 8618 (12.9%) patients and type 2 MI in 382 of 8,618 (4.4%) patients. In sum, the primary diagnostic endpoint occurred in 1491 of 8618 (17.3%) patients. The frequency of other diagnoses is shown in the Online Table 4.

The median follow-up was 821 (interquartile range 812 to 832) days. During this follow-up, 931 of 8618 (11.1%) patients died in the total population. Of these, 51 deaths (0.6%) occurred while the patient was in-hospital and 202 (2.4%) deaths occurred within 6 months of enrollment. Details on risk discrimination according to the *original GRACE risk score* and the ESC 0/1 h-algorithm are shown in Online Fig. 2.

### Impact on clinical management

The positive predictive value (PPV) for MI of the ESC 0/1 h-algorithm rule-in pathway was 74.5% (95% CI 72.2 to 76.6%) with a specificity of 94.4% (95% CI 93.8–94.9%) and a sensitivity of 78.1% (95% CI 75.9–80.2%).

**Fig. 1:**
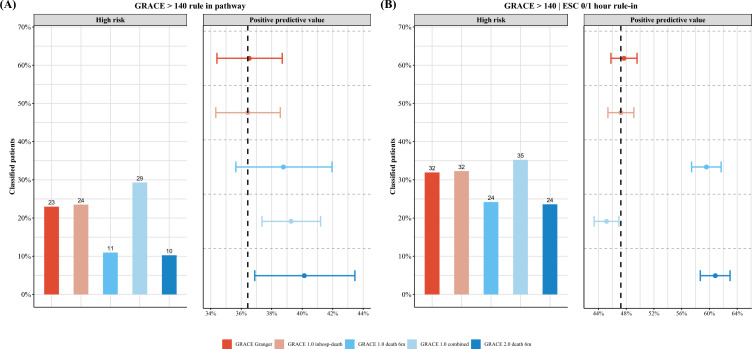
Impact of GRACE risk score choice on the proportion of patients considered to be at high risk. Proportion of patients considered to be at high risk according to chosen GRACE risk score (bar plot) with corresponding positive predictive value for the final diagnosis of myocardial infarction (dot plot with 95% confidence interval). *ESC* European Society of Cardiology

The PPV for MI of a score > 140 in the *original GRACE risk score* was 36.5% (95% CI 34.4–38.7%) with a specificity of 82.4% (95% CI 81.5–83.2%) and a sensitivity of 48.5% (95% CI 45.9–51.1%). The PPVs of other GRACE risk scores considering a longer follow-up than the index hospitalization were statistically higher but this is likely without clinical significance. Clinically more significant will be differences in the sensitivity between the GRACE risk scores (Fig. 1a; Table 3).

**Table 3:**
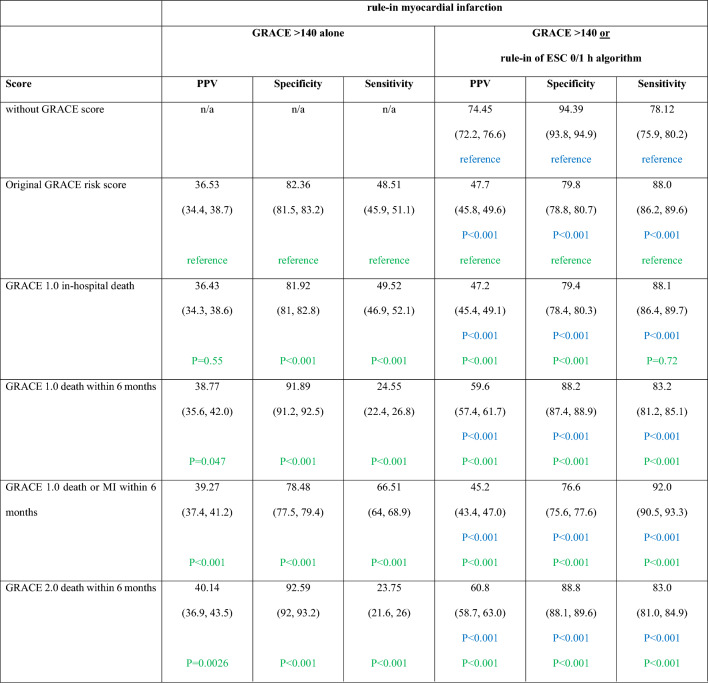
Comparison of GRACE risk score models in combination with the ESC 0/1 h-algorithm or alone in regard to guideline-recommended criteria for an early invasive strategy

Impact of the GRACE risk scores alone or in combination with the ESC 0/1 h-algorithms (rule-in category) on the diagnostic performance to identify patients with myocardial infarction. Presented data are based on imputed data

Blue highlighted *P* values relate to tests comparing against the rule-in category of the ESC 0/1 h-algorithm

Green highlighted *P* values relate to tests comparing against the original GRACE risk score estimating the risk for in-hospital death

*MI* myocardial infarction, *n/a* not applicable, *PPV* = positive predictive value

The combination of the ESC 0/1 h-algorithm rule-in pathway with the GRACE risk score criterion > 140 led to increases in sensitivity but decreased specificity and PPV (Fig. 1b; Table 3).

**Fig. 2:**
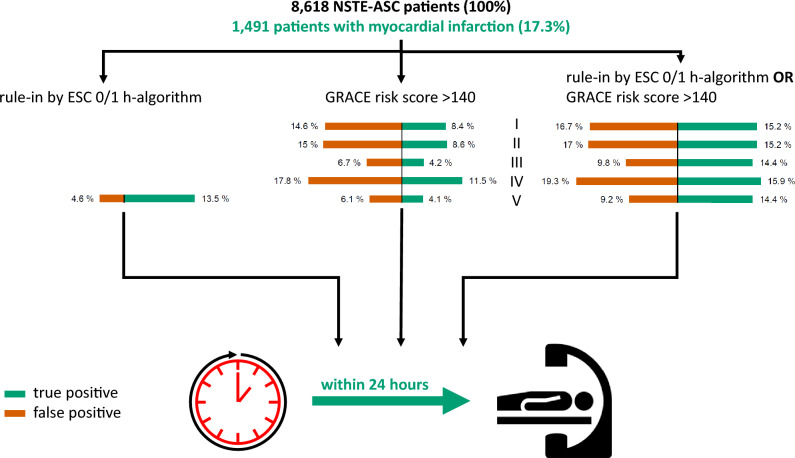
Impact of definition of high ischemic risk on the proportion of patients qualifying for an early invasive strategy. The study population comprises 8618 NSTE-ACS patients. Of those, 1491 patients (17.3%) had a myocardial infarction (MI). The ability of the guideline-recommended criteria for an early invasive strategy to identify patients with a MI considerably differs as depicted in the figure. All patients with a positive test (red bar plus green bar) qualify for an early invasive strategy. However, the positivity of the test can be either false (red bar) or true (green bar), i.e., the patient does not have MI or does have MI, respectively. While the ESC 0/1 h-algorithm (left bar plot) results in considerably more true positive (TP) than false positive (FP) results, the ratio is the other way around for the GRACE risk scores (middle column of bar plots). The proportion of missed MIs by the test (i.e., false negatives, FN) is the amount of patients with an MI (17.3%) minus the TPs. Combining the ESC 0/1 h-algorithm with a GRACE risk score to a single test (right column of bar plots) increases the proportion of TPs but at the cost of a higher number of FP test results. Test results also differ clinically relevant between the difference GRACE risk scores: I = original GRACE risk score estimating the risk for in-hospital death; II = GRACE 1.0 estimating the risk for in-hospital death; III = GRACE 1.0 estimating the risk for death at 6 months; IV = GRACE 1.0 estimating the combined risk for death or myocardial infarction at 6 months; V = GRACE 2.0 estimating the risk for death at 6 months

According to the European guideline recommendation, patients qualify for an early invasive strategy if they are in the rule-in category of ESC 0/1 h-algorithm or have GRACE risk score > 140. Such patients are either true positives (i.e., have MI) or false positives (i.e., do not have MI). The number of patients qualifying for an early invasive strategy as well as the distribution of true and false positive test results considerably varied depending on the applied test (Online Table 6, Fig. 2).

### Prediction of NSTEMI

In logistic regression models, rule-in triage of the ESC 0/1 h-algorithm (odds ratio 60.08, 95% CI 51.16–70.56, *p* < 0.001) as well as a score > 140 in the or*iginal GRACE risk score estimating the risk for in-hospital death* (odds ratio 4.40, 95% CI 3.91–4.95, *p* < 0.001) predicted well a final NSTEMI diagnosis. Both covariates remained significantly associated in a multivariable model. The predictive ability of the multivariable model was better than both single variable models as tested by means of the log-likelihood ratio test. The application of other GRACE risk scores led to very similar results (Online Table 7).

## Discussion

In this large prospective, international, multicenter study using central adjudication, the impact on guideline-recommended treatment decisions between different GRACE risk scores was compared among each other and with the ESC 0/1 h-algorithm. There are three major findings: First, using GRACE risk scores other than the *original GRACE risk score* leads to substantial reclassification of the estimated risk if the same cutoffs (i.e., ≤ 108 and > 140) were used. Second, the ability to detect patients with MI (i.e., sensitivity) differs considerably among the ESC 0/1 h-algorithm and GRACE risk scores with the best performance of a single test by the ESC 0/1 h-algorithm. Third, supplementing the ESC 0/1 h-algorithm with a GRACE risk score improves the sensitivity of diagnosing MI in a statistically significant way and clinically relevant way, but at the cost of an increase in false positive test results.

The TIMACS trial compared an early invasive strategy (coronary angiography within 24 h of randomization) with a delayed invasive strategy (coronary angiography after 36 h of randomization) in patients presenting with NSTE-ACS [8]. There was no difference between the two strategies on the primary endpoint of death, MI, or stroke at 6 months in the overall population. A pre-specified subgroup analysis showed that an early invasive strategy improved the primary endpoint in the highest risk tertile according to the GRACE risk score (i.e., > 140) but not in the two thirds at low-to-intermediate risk (≤ 140, p-interaction = 0.01) [8]. Based on this subgroup analysis, the ESC NSTE-ACS 2011 guidelines recommended an early invasive strategy within 24 h for patients with a GRACE score > 140 [13]. For this use, the GRACE risk score went beyond its original role for risk stratification and was recommended to guide treatment decisions. Moreover, this recommendation was carried forward in subsequent guidelines. Unfortunately, most guidelines do not specify which GRACE risk score should be calculated. The only exception are the American 2021 guidelines for coronary artery revascularization, which specifically recommend the *GRACE 2.0 estimating the risk for death at 6 months* for the timing of invasive coronary angiography. However, the cutoff of > 140 for the *original GRACE risk score* indicates a risk of > 3% for in-hospital death and does not necessarily indicate also a clinically meaningful increased risk for other GRACE risk scores.

The application of other GRACE risk scores reclassified a considerable number of patients using the cutoffs of the *original GRACE risk score* in our population. Reclassification was especially prominent in the GRACE 1.0 and GRACE 2.0 scores estimating the risk for death within 6 months. Here, more than 50% of patients were downgraded from intermediate to low or high to intermediate risk. The latter is of particular importance in light of the study aim (2), since these downgraded patients would no longer qualify for an early invasive strategy according to guideline recommendations.

A GRACE risk score > 140 is the only criterion in the American and one of the criteria in the ESC guidelines, respectively, defining a high ischemic risk for which an early invasive strategy within 24 h is recommended [5, 6, 13]. Considering the large proportion of reclassification, the question is raised to which amount treatment decisions based on this recommendation are influenced by the choice of the GRACE risk score.

As outlined above, this recommendation is based on a subgroup analysis of the neutral TIMACS trial, in which the *original GRACE risk score* was used. The VERDICT trial used also the *original GRACE risk score* and further supports the use of the GRACE risk score for invasive coronary angiography timing. VERDICT—independently from TIMACS—showed a benefit of an early invasive strategy in patients with a GRACE risk score > 140 while no such benefit was evident in patients with a GRACE risk score ≤ 140 or the overall population [14]. This finding also appeared to be true with regard to all-cause mortality in a post hoc analysis [15]. These VERDICT analyses suggest that the GRACE risk score is the key player for guiding the timing of invasive coronary angiography in NSTE-ACS.

Patients with the final diagnosis of MI represent the subset of the NSTE-ACS population with the presumed highest benefit from an early invasive strategy. Thus, although the GRACE risk score was not developed as a diagnostic tool, and in fact was originally developed on populations already diagnosed with ACS, the proportion of patients with high risk based on GRACE risk scores who have MI is relevant. Our analyses revealed that the PPV for MI detection is only 36.5% for the *original GRACE risk score.* GRACE risk scores predicting the outcome after a longer follow-up (the risk for in-hospital death versus the risk for death at 6 months or the combined risk for death or myocardial infarction at 6 months) had a significantly higher but still only modest PPV for MI diagnosis with the highest of 40.0% for the *GRACE 2.0 estimating the risk for death at 6 months*. However, all these PPVs are dramatically lower than the 74.5% PPV for the ESC 0/1 h-algorithm, which was developed for the detection of MI. Moreover, sensitivity to detect MI of the ESC 0/1 h-algorithm is much higher than for any GRACE risk score. Therefore, the ESC 0/1 h-algorithm rule-in category based on hs-cTn poses the more accurate gatekeeper for an early invasive strategy as recommended in the ESC guidelines which is in contrast to recent American guidelines [6, 7].

Expressed differently, considerably fewer patients with an acute MI would be sent to invasive coronary angiography within 24 h by relying the decision on a GRACE risk score > 140 as compared to the ESC 0/1 h-algorithm if these had no indication for an immediate approach (i.e., refractory angina, hemodynamic instability, or cardiogenic shock) [7]. Moreover, the American guideline specifically recommends the *GRACE 2.0 estimating the risk for death at 6 months* for timing of invasive coronary angiography. Even though the PPVs are similar, the sensitivity to detect MI by the *GRACE 2.0 estimating the risk for death at 6 months* is the lowest of all GRACE risk scores. *GRACE 1.0 estimating the combined risk for death or MI* would detect almost three times more MIs.

Combining the ESC 0/1 h-algorithm with a GRACE risk score to one test significantly increases the test’s ability to detect MI (i.e., sensitivity). This, however, is associated with increasing rates of false positive results, whereby the PPV decreases. Higher numbers of false positive test results might expose those patients to the risk for complications as a consequence of unnecessary invasive coronary angiography, assuming those with high GRACE risk score but without contemporary biomarker evidence of MI will derive less benefit. Clinicians have to decide this tradeoff by balancing the risk of a missed or later detected MI with the risk for procedural complications.

No randomized controlled trial investigating the optimal timing of invasive coronary angiography in NSTE-ACS used dynamic troponin changes measured by high-sensitivity assays as inclusion criterion or for a predefined subgroup analysis. Conflicting data exists for the GRACE risk score. While the results of the Australian GRACE Risk Score Intervention Study (AGRIS) apparently argue against the application of the GRACE risk score criterion [16], the independent subgroup findings of TIMACS and VERDICT are in favor of it [8, 14]. Moreover, the benefit of an early invasive strategy observed for NSTE-ACS patients with a GRACE risk score > 140 in TIMACS and VERDICT might become more or less relevant if only patients in the rule-in category of the ESC 0/1 h-algorithm would be recruited. Nevertheless, risk discrimination by established GRACE risk score which were studied in this analysis is good as documented multiple times [1, 2, 8, 14, 17]. This performance was even improved in GRACE 3.0 using machine learning models [18]. This new score directly depicts the risk for in-hospital death in percent and not cumbersomely as point score (e.g., > 3% rather than > 140). However, no scientific evidence for the GRACE 3.0’s ability to identify patients profiting from an early invasive strategy currently exists yet.

### Limitations

First, the Killip class was not prospectively assessed in both APACE and BACC. We, therefore, approximated the Killip class based on the NYHA class and systolic blood pressure on admission. This resulted in a similar distribution of Killip classes in comparison to the derivation cohort of the *original GRACE risk score* [1].

Second, some variables necessary to calculate GRACE risk scores were missing. However, the proportion of missing values was low. Therefore, multiple imputation was used to handle missing data. By this, 2.2% of patients could be retained in the analyses, which would have been excluded in a complete case analysis. Therefore, multiple imputation typically improves precision of estimates unless the mechanisms of missing is missing not at random which is not likely in our dataset [19].

Third, although we used the most stringent methodology to adjudicate the presence or absence of NSTEMI including central adjudication by experienced cardiologists and serial measurements of hs-cTnT, we still may have misclassified a small number of patients [20, 21].

Fourth, we cannot generalize our findings to patients with end-stage kidney failure requiring dialysis, since these patients were excluded from this study.

## Conclusion

Using GRACE risk scores other than the *original GRACE risk score* leads to substantial reclassification of the estimated risk. The proportion of identified patients with MI (i.e., sensitivity for diagnosis of MI) dramatically differs between the GRACE risk scores. The ability of the ESC 0/1 h-algorithm to identify patients with confirmed MI is far superior to any GRACE risk score alone. Supplementing the ESC 0/1 h-algorithm by the criterion GRACE risk score > 140 improves the sensitivity to identify patients with MI that on the other hand leads to more false positives. Therefore, the preferred approach may be the use of the ESC 0/1 h-algorithm without the GRACE score, though randomized trials are necessary to determine the best algorithm to determine which patients most benefit from an early invasive approach.

## Data Availability

The data that support the findings of this study are not openly available due to reasons of sensitivity. Data are located in controlled access data storage at the University Hospital Basel as well as Hamburg.
